# Predictive Factors of the Degrees of Malnutrition According to GLIM Criteria in Head and Neck Cancer Patients: Valor Group

**DOI:** 10.3390/cancers16244255

**Published:** 2024-12-21

**Authors:** Francisco Javier Vílchez-López, María González-Pacheco, Rocío Fernández-Jiménez, María Teresa Zarco-Martín, Montserrat Gonzalo-Marín, Jesús Cobo-Molinos, Alba Carmona-Llanos, Araceli Muñoz-Garach, Pedro Pablo García-Luna, Aura D. Herrera-Martínez, Felisa Pilar Zarco-Rodríguez, María del Carmen Galindo-Gallardo, Luis Miguel-Luengo, María Luisa Fernández-Soto, José Manuel García-Almeida

**Affiliations:** 1 Department of Endocrinology and Nutrition, Instituto de Investigación e Innovación Biomédica de Cádiz (INIBICA), Puerta del Mar University Hospital, 11009 Cadiz, Spain; franciscoj.vilchez.sspa@juntadeandalucia.es (F.J.V.-L.); maria.gonzalez@inibica.es (M.G.-P.); 2 Department of Endocrinology and Nutrition, Virgen de la Victoria University Hospital, Málaga Biomedical Research Institute and BIONAND Platform (IBIMA), 29010 Malaga, Spain; jgarciaalmeida@uma.es; 3 Department of Endocrinology and Nutrition, QuironSalud Malaga Hospital, 29004 Malaga, Spain; 4 Department of Medicine and Dermatology, Faculty of Medicine, University of Malaga, 29010 Malaga, Spain; 5 Department of Endocrinology and Nutrition, Hospital Universitario Clínico San Cecilio, Granada, Biosanitary Research Institute of Granada (ibs.GRANADA), 18012 Granada, Spain; terezarco@correo.ugr.es; 6 Department of Endocrinology and Nutrition, Regional de Málaga University Hospital, Málaga Biomedical Research Institute and BIONAND Platform (IBIMA), 29010 Malaga, Spain; montsegonzalo@yahoo.es; 7 Clinical Trials, Jaén University Hospital/FIBAO, 23007 Jaén, Spain; jcobos@ujaen.es; 8 Department of Endocrinology and Nutrition, Instituto de Investigación e Innovación Biomédica de Cádiz (INIBICA), Jerez de la Frontera University Hospital, 11407 Cadiz, Spain; alba.carmona@inibica.es; 9 Department of Endocrinology and Nutrition, Hospital Universitario Virgen de las Nieves, Granada, Biosanitary Research Institute of Granada (ibs.GRANADA), 18012 Granada, Spain; araceli.munoz.garach.sspa@juntadeandalucia.es; 10 Centro de Investigación Biomedica en Red Fisiopatologia de la Obesidad y Nutrición(CiberOBN), Instituto de Salud Carlos III, 28029 Madrid, Spain; 11 Department of Endocrinology and Nutrition, Virgen del Rocío University Hospital, Instituto de Biomedicina de Sevilla (IBIS), 41013 Seville, Spain; pgarcia1@us.es; 12 Department of Endocrinology and Nutrition, Reina Sofía University Hospital, Maimonides Institute for Biomedical Research of Cordoba (IMIBIC), 14004 Córdoba, Spain; aurad.herrera.sspa@juntadeandalucia.es; 13 Department of Endocrinology and Nutrition, Valme University Hospital, 41014 Seville, Spain; felisa.zarco.sspa@juntadeandalucia.es; 14 Department of Endocrinology and Nutrition, Macarena University Hospital, Fundación Pública Para la Gestión de Salud en Sevilla (FISEVI), 41009 Seville, Spain; mariac.galindo@juntadeandalucia.es; 15 Department of Biomedical Sciences, Endocrinology and Nutrition Service, Badajoz University Hospital, Universidad de Extremadura, 06080 Badajoz, Spain; luismluengo@unex.es; 16 Endocrinology and Nutrition Clinical Management Unit, University Hospital San Cecilio, 18012 Granada, Spain; 17 Biosanitary Institute of Granada, Medicine Department, Faculty of Medicine of Granada, University of Granada, 18010 Granada, Spain

**Keywords:** head and neck cancer, glim criteria, malnutrition, morphofunctional assessment

## Abstract

**Background:** Malnutrition is highly prevalent in patients with head and neck cancer, with relevant consequences in the treatment results. **Methods:** Multicenter observational study including 514 patients diagnosed with HNC. The morphofunctional assessment was carried out during the first 2 weeks of radiotherapy treatment. A correlation analysis between nutritional variables and groups of malnutrition, a multivariate logistic regression analysis, and a random forest analysis to select the most relevant variables to predict malnutrition were performed. **Results:** In total, 51.6% were undernourished (26.3% moderately and 25.3% severely). There was a negative correlation between morphofunctional variables and a positive correlation between hsCRP and well vs. moderate and well vs. severe malnutrition groups. The increase in different bioelectrical and ultrasound parameters was associated with a lower risk of moderate and severe malnutrition when groups with different degrees of malnutrition were compared. To predict the importance of morphofunctional variables on the risk of undernutrition, a nomogram, a random forest, and decision tree models were conducted. For the well vs. moderate, for the well vs. severe, and for the moderate vs. severe malnutrition groups, FFMI (cut-off < 20 kg/m^2^), BCMI (cut-off < 7.6 kg/m^2^), and RF-Y-axis (cut-off < 0.94 cm), respectively, were the most crucial variables, showing a greater probability of mortality in the two last comparisons. **Conclusions:** Malnutrition is very prevalent in HNC patients. Morphofunctional assessment with simple tools such as electrical impedance and muscle ultrasound allows an early nutritional diagnosis with an impact on survival. Therefore, these techniques should be incorporated into the daily clinical attention of patients with HNC.

## Simple Summary

Patients with head and neck cancer (HNC) have a high prevalence of malnutrition, which is associated with a decrease in overall survival. In this study, we carried out a morphofunctional assessment (bioelectrical impedance, muscle ultrasound, hand grip strength, the up-and-go test, and biochemical analysis) in a population with HNC. We detected a correlation between malnutrition and morphofunctional measurement. Multiple bioelectrical and ultrasound parameters were associated with an increased risk of malnutrition and its severity. We found, for the first time in this population, different cut-off points to define malnutrition based on Glim Criteria and its severity, showing a greater probability of mortality when comparing severe malnutrition group versus well and moderate malnutrition, respectively. Therefore, with simple techniques such as BIA and ultrasound, an individualized diagnosis of malnutrition can be reached, so they should be incorporated into the daily clinical practice to identify higher-risk patients in whom to intensify nutritional treatment.

## 1. Introduction

In 2020, approximately 880,000 patients with head and neck cancer (HNC) were diagnosed worldwide, resulting in 445,000 deaths, with an estimated 5 year survival rate of 50% [1]. Due to the current therapy, patients with HNC frequently experience toxicity, which has a significant nutritional impact, resulting in malnutrition [2]. Consequently, malnutrition in patients with HNC decreases the efficacy of therapy [3,4], quality of life [3,5], and survival [4,6], as well as increases the risk of infection, postoperative complications, and readmissions [3,7,8]. Up to 70% of weight loss in cancer patients is attributed to the reduction of muscle mass, which, specifically in patients with HNC, has been associated with decreased survival [9,10,11,12]. Therefore, early detection of malnutrition and further immediate nutritional intervention is crucial to optimize oncological treatment outcomes [13,14].

Nonetheless, the prevalence of malnutrition in patients with HNC ranges from 20% to 74%, depending on the tumor location, disease stage, as well as the criteria used to define malnutrition [15,16,17,18,19,20,21]. Even when applying the previous diagnostic criteria of societies such as the American Society for Parenteral and Enteral Nutrition (ASPEN) and The European Society for Clinical Nutrition and Metabolism (ESPEN), significant differences of up to 40% in the prevalence of malnutrition are observed [22]. For these reasons, it is difficult to compare the results of different studies with each other.

Therefore, The GLIM (Global Leadership Initiative on Malnutrition) criteria have been introduced to unify diagnosis for malnutrition, including the assessment of muscle mass and inflammation [23]. GLIM criteria have been validated in different studies concerning patients with HNC [21,24,25,26], although there was a high variable prevalence, from 3.7% to 57% [21,24,27,28,29], due to discrepancies between combinations of phenotypic and etiologic criteria used, as well as different criteria for defining inflammation and muscle mass reduction [30]. However, until now, CT at the L3 level (along with DEXA) is considered the gold standard technique for assessing muscle mass, although this technique is not intentionally used for this purpose [31]. This is why, in recent years, there has been a growing interest in developing new techniques, such as bioelectrical impedance analysis and nutritional ultrasound, that are simple, non-invasive, reproducible, and can be very useful for the periodic assessment of the nutritional status of patients with HNC, in whom this status usually fluctuates. Results published by our group show that a BCMI < 7.8 predicts the diagnosis of malnutrition. Mortality was higher in the malnourished group [32]. In addition, different bioelectrical parameters derived from BIA (PA, SPA, BCM, and BCMI) behave as prognostic factors in patients with head and neck cancer, presenting PA values < 5.1° in male and <4.8° in female patients as the best predictive potential for mortality [33]. However, it is often difficult to establish the degree of malnutrition on the basis of the GLIM criteria, as there are no normal values defining the reduction in muscle mass. For this purpose, the objective of our study is to describe not only the prevalence of malnutrition but also the prevalence of each degree of undernutrition in a series of patients with HNC during the first 2 weeks of radiotherapy, to detect parameters derived from morphofunctional assessment that are predictive of each malnutrition grade according to the GLIM criteria, as well as to correlate the malnutrition grade and the overall survival rates, to finally define new nutritional tools to predict early malnutrition and related consequences in patients with HNC.

## 2. Materials and Methods

### 2.1. Study Design

Observational longitudinal study conducted by the group “VALOR: VALoracion morfofuncional en el paciente OncoRadioterápico” in the Nutrition Units of 12 hospitals in Andalusia, Spain [Hospital Universitario “Virgen de la Victoria” and Hospital Regional de Málaga (Málaga), Hospital Universitario de Jaén (Jaén), Hospital “San Cecilio” and Hospital Universitario “Virgen de las Nieves” (Granada), Hospital Universitario “Puerta del Mar” and Hospital Universitario de Jerez de la Frontera (Cádiz), Hospital Universitario Virgen del Rocío, Hospital Universitario Virgen Macarena and Virgen de Valme (Sevilla), Hospital Universitario Reina Sofía (Córdoba), and Hospital Universitario de Badajoz (Badajoz)] between 2020 and 2022. The study was approved by the Ethics Committee of Hospital Universitario “Virgen de las Nieves” (reference code: 2381-M1-22).

The study included 514 patients diagnosed with HNC at different stages and treated with radiotherapy. Morphofunctional assessment was performed just before or during the first two weeks of treatment (whether or not associated with chemotherapy or other systemic treatments). The radiotherapy treatment could modify the nutritional status in some cases, but this fact did not affect the main objective of our study. The diagnosis was confirmed through clinical histories and pathological examinations, and biopsy samples were classified by pathologists according to histological characteristics following the guidelines of the “World Health Organization Classification of Tumors of the Digestive System” (2019) [34]. A minimum follow-up of 12 months was conducted, with visits at 3, 6, and 12 months during the first year. All patients agreed to participate by signing an informed consent form. Patients who refused to undergo nutritional measurements using BIA due to reasons such as ethnicity, extensive skin lesions, fluid extravasation, local hematomas, amputation, or having a life expectancy of less than 3 months were excluded.

### 2.2. Demographic, Clinical, and Biochemical Variables

Included age (years), sex (male/female), BMI (kg/m^2^), weight (kg), and weight loss (%), TNM stage [1,2,3,4], chemotherapy (yes/no), complications (yes/no): dermatitis, dysphagia, mucositis, asthenia, unplanned admissions, disease persistence/disease-free, and death. We used the Eastern Cooperative Oncology Group (ECOG) to assess functional status and quality of life (0: asymptomatic, normal activity, 1: symptomatic, ambulatory, 2: bedridden <50% of the day, minimal assistance, 3: bedridden >50% of the day, assistance, 4: bedridden all day, severely limited, 5: deceased). Levels of albumin (g/dL), prealbumin (mg/dL), proteins (g/dL), total cholesterol (mg/dL), creatinine (mg/dL), glomerular filtration rate (mL/min), urea (mg/dL), glucose (mg/dL), C-reactive protein (CRP, mg/dL), TSH (µUI/mL), and HbA1c (%) were determined.

### 2.3. Morphofunctional Assessment

A body composition study was conducted using bioelectrical impedance analysis (BIA) and nutritional ultrasound. Muscle strength was determined using dynamometry, and functionality was assessed using the Up and Go test. The bioelectrical impedance analysis was performed with a 50 kHz phase-sensitive impedance analyzer (BIA 101 Whole Body Bioimpedance Vector Analyzer, AKERN, Florence, Italy) with tetrapolar electrodes emitting 800 μA. The electrodes were placed on the right hand and foot, and measurements were taken with the patient in a supine position after 5 min of rest to stabilize fluid displacement. Phase angle (PA, °), standardized phase angle (SPA), body cell mass (BCM, kg), body cell mass index (BCMI, kg/m^2^), fat mass (FM, kg), fat mass index (FMI, kg/m^2^), fat-free mass index (FFMI, kg/m^2^), appendicular skeletal muscle mass (ASMM, kg), skeletal muscle index (SMI, kg/m^2^), total body water (TBW, kg), extracellular water (ECW, kg), intracellular water (ICW, kg), hydration levels (%), reactance (Xc, Ω/m), and resistance (Rz, Ω/m) values were obtained. Height was measured using a Seca stadiometer (Hamburg, Germany). BIA measurements of patients were standardized by sex and age using data from healthy Italian adults [35]. The PA is expressed in degrees as arctan (Xc/Rz) × (180°/π). The standardized PA (SPA) value for each individual was determined from the reference population value by subtracting the reference PA value from the observed PA value in the patient and dividing the result by the respective reference standard deviation (SD) for age and sex [36]. The precision of the BIA instrument was evaluated daily using a precision circuit provided by the BIA device manufacturer (AKERN, Florence, Italy), consistently showing values close to the reference value of 385 Ohms. The in vivo reproducibility of the BIA measurements showed coefficients of variation (CV) of 1–2% for Rz and Xc.

We performed a muscle ultrasound of the rectus femoris of the quadriceps (QRF) using a 10–12 MHz probe and a multifrequency linear array (Mindray Z60, Madrid, Spain) in all subjects in a supine position. The evaluation was performed without compression at the lower third between the superior pole of the patella and the anterior superior iliac spine, measuring the anteroposterior muscle thickness, circumference, and cross-sectional area [37]. The ultrasound was performed by a specific physician previously trained in this technique. The probe was aligned perpendicularly to the longitudinal and transverse axes on the QRF to measure the cross-sectional area of the rectus femoris (RF-CSA), the circumference of the rectus femoris (RF-CIR), the RF axes (X-axis and Y-axis), and the leg subcutaneous fat (L-SAT). Three measurements were taken for each parameter, and the mean was calculated. For the evaluation of adipose tissue in the abdominal area, the midpoint between the xiphoid process and the navel was measured, where total subcutaneous abdominal fat (T-SAT), superficial subcutaneous abdominal fat (S-SAT), and preperitoneal or visceral fat (VAT) were measured in centimeters. Global adipose tissue (GAT) and the GAT index (GATi) were calculated by summing T-SAT, L-SAT, and VAT and dividing the result by the height, respectively. To measure handgrip strength (HGS), we used a Jamar hand dynamometer (Asimow Engineering Co., Los Angeles, CA, USA). Handgrip strength was measured in a seated position with the elbow flexed at 90° in the dominant hand. Patients were instructed to perform three maximal isometric contractions with brief pauses between measurements, and the maximum and average values were recorded. Functional capacity was assessed using the Timed Up and Go test, measuring the time in seconds it took to get up from a chair, walk 3 m, turn around, walk another 3 m, and sit back down.

### 2.4. Nutritional Diagnosis

To establish the diagnosis of malnutrition according to GLIM criteria [23], the combination of a phenotypic criterion and an etiologic criterion was required. The phenotypic criteria used for the diagnosis of moderate cases were: weight loss between 5% and 10% in the last 6 months, BMI < 20 kg/m^2^ in those under 70 years, or BMI < 22 kg/m^2^ in patients aged 70 years or older, or a FFMI < 17 kg/m^2^ in men or <15 kg/m^2^ in women. Severe cases were considered those with weight loss greater than 10%, with BMI < 18.5 kg/m^2^ and age < 70 years or <20 kg/m^2^ and age ≥ 70 years. The etiologic criteria used were reduced food intake/assimilation and disease/inflammation burden. Reduced intake was considered when it represented less than 75% of the calculated needs after conducting a 24 h intake recall. The presence of gastrointestinal symptoms, such as dysphagia, nausea, vomiting, diarrhea, constipation, and abdominal pain, was investigated as indicators of poor food assimilation. In the presence of an active tumor requiring radiotherapy treatment, it was considered that all patients included in the study met the etiologic criterion of inflammation [30].

The main clinical outcomes were the mortality rate at 600 days, complications arising from RT treatment (dermatitis, dysphagia, mucositis, and asthenia), the need for hospitalization during or after treatment, or the need for palliative care (when the disease was in a terminal phase with no possibility of cure with conventional treatments). Using physical examination and imaging tests, disease-free status was defined as tumor remission after initial therapy in imaging tests. Disease persistence was considered if the cancer remained stable, and disease progression was defined as tumor worsening (local or distant). Disease status was established by the responsible physician, and clinical outcomes were extracted from the hospital medical record.

### 2.5. Statistical Analysis

The results are presented as mean ± standard deviation (SD) for continuous variables and as numerical values (percentages) for categorical variables. The asterisk indicates a significant difference between groups, according to the ANOVA test, followed by pairwise comparisons adjusting for multiple testing (Benjamini & Hochberg method). The chi-squared test was used for variables expressed as percentages. Some variables, such as Cancer stage, Complications, and Mortality, include missing data (NA values) due to incomplete records for certain patients. These missing values account for the discrepancies observed between the total sample size (N) and the sum of these variables across nutritional status categories. Statistical analyses were performed, considering these missing data as appropriate. Through logistic regression analysis to assess the association of different body composition and functionality parameters with the degree of malnutrition, the odds ratio (OR) (95% confidence intervals [CI]) was obtained. The results were adjusted for age, sex, and BMI (Categorized as BMI less than 20 for patients aged under 70 and BMI less than 22 for patients aged 70 or older). The predictive property assessment of muscle mass variables for malnutrition was based on the receiver operating characteristic (ROC) curve and the AUC. We used multivariable COX regression to predict the risk of mortality based on the results of an analysis using the rms package and the foreign package of the R software. A decision tree was created using the rpart package, a random forest was implemented using the RandomForest package analyses, and the graphical representation was performed with R software v. 3.5.1 (Integrated Development for R. RStudio, PBC, Boston, MA, USA). Statistical significance was considered with a *p*-value < 0.05.

## 3. Results

### 3.1. General Characteristics

The study included 514 patients with HNC categorized based on their nutritional status into well-nourished (N = 249; 48.4%), moderate (N = 135; 26.3%), and severe (N = 130; 25.3%) malnourished groups. General characteristics are summarized in Table 1. Overall, patients with severe and moderate malnutrition had decreased BMI and BIA variables when compared with well-nourished patients (*p* < 0.05). Additionally, patients with severe malnutrition had decreased muscle mass, measured by ultrasound tools, such as RF-CSA (Rectus Femoris Cross Sectional Area) and -Circumference, X- and Y-axis, when compared with moderate and well-nourished patients (*p* < 0.05). Finally, patients with severe and moderate malnutrition have decreased muscle strength, measured by the hand grip tool, when compared with well-nourished patients (*p* < 0.05). Supplementary Table S1 shows additional data about other values, such as BIA, muscle mass and water content variables, and other clinicopathological variables. Briefly, patients with severe and moderate malnutrition have decreased BIA, muscle mass, and water content variables when compared with well-nourished patients (*p* < 0.05).

### 3.2. Morphofunctional Assessment in the Diagnosis of Malnutrition

**Figure 1:**
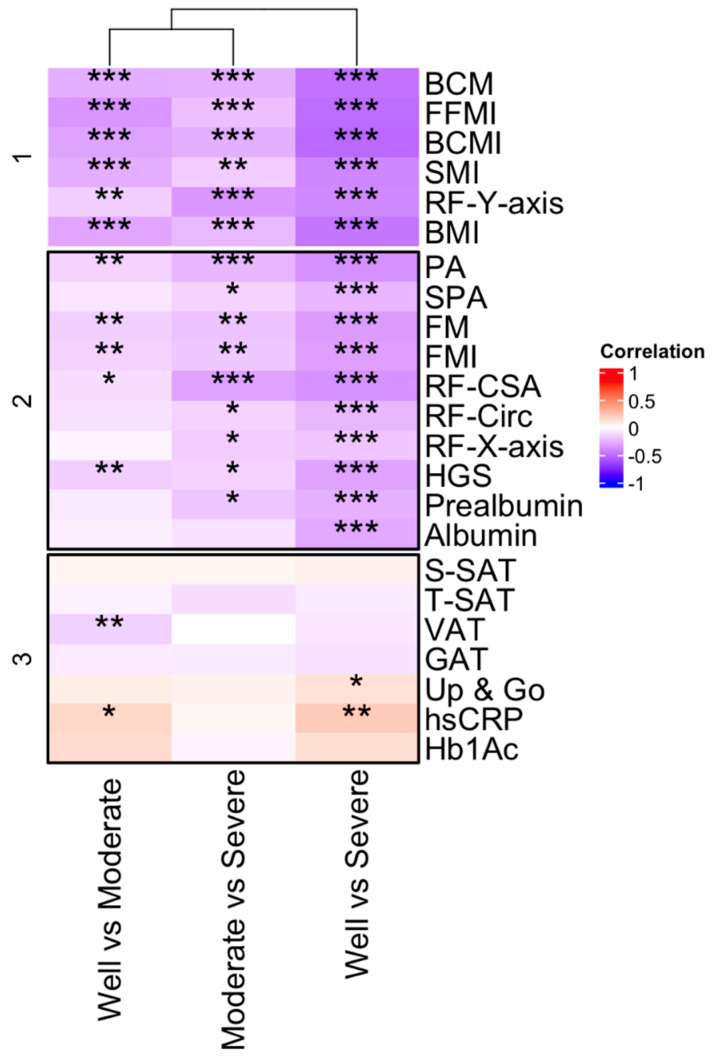
Significant correlations between body composition parameters assessed by BIVA and ultrasound nutritional evaluation, biochemical nutritional parameters, and sarcopenia (* *p* < 0.05; ** *p* < 0.01; *** *p* < 0.001). BIVA: Bioelectrical impedance vector analysis.

To test which morphofunctional measurement was suitable to differentiate the grade of malnutrition (Well vs. moderate; Moderate vs. severe; Well vs. Severe), a morphofunctional assessment was performed by comparing the three groups. To do this, a biserial correlation was conducted to evaluate the correlation between quantitative and binary variables (groups of malnutrition) (Figure 1). When we focused on all patients, we generally found a negative correlation between morphofunctional variables and groups (almost all with *p* < 0.001). In contrast, we found a positive correlation between hsCRP and well vs. moderate and well vs. severe groups (*p* < 0.05 and *p* < 0.01, respectively). Supplementary Figure S1 and Supplementary Figure S2 are summarized in Figure 1, separated by males and females, respectively.

Furthermore, we assessed a multivariate logistic regression analysis, adjusted for age, sex, and BMI, to evaluate the relationship between malnutrition and morphofunctional measurement (Table 2). As for the well vs. moderate malnutrition group, we found that increased BCM, FFMI, BCMI, SMI, and RF-Y-axis were associated with decreased risk of developing moderate malnutrition, with an OR of 0.89 (95% CI: 0.84–0.94), 0.70 (95% CI: 0.61–0.80), 0.69 (95% CI: 0.58–0.81), 0.56 (95% CI: 0.43–0.70) and 0.44 (95% CI: 0.21–0.87), respectively. As for the well vs. severe malnutrition group, we found that increased PA, SPA, BCM, FFMI, BCMI, SMI, RF-CSA and RF-Y-axis were associated with decreased risk to develop severe malnutrition, with an OR of 0.48 (95% CI: 0.33–0.69), 0.70 (95% CI: 0.53–0.91), 0.85 (95% CI: 0.79–0.91), 0.71 (95% CI: 0.59–0.84), 0.6 (95% CI: 0.47–0.73), 0.56 (95% CI: 0.41–0.75), 0.65 (95% CI: 0.50–0.84) and 0.14 (95% CI: 0.05–0.36) respectively. Finally, as for the moderate vs. severe malnutrition group, we found RF-CSA and RF-Y-axis were associated with a decreased risk of developing severe malnutrition, with an OR of 0.66 (95% CI: 0.49–0.87) and 0.16 (95% CI: 0.05–0.45), respectively.

### 3.3. Machine Learning and Decision Tree to Select the Most Important Variable to Predict Malnutrition in Patients with HNC

**Figure 2:**
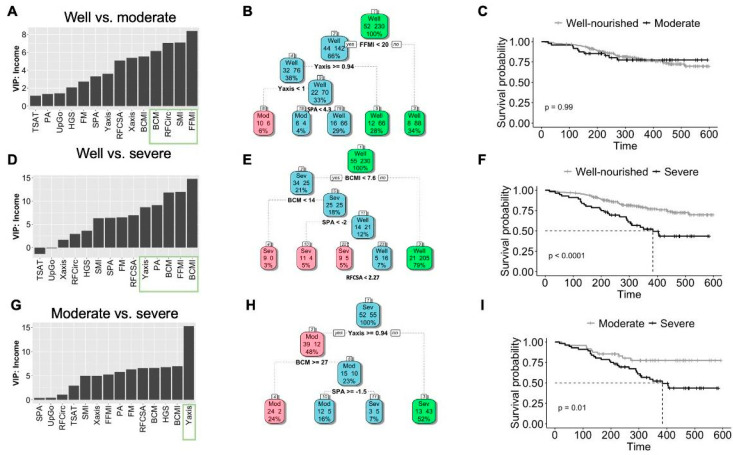
Clinical algorithm for predicting malnutrition in HNC patients using body composition parameters determined by BIVA ultrasound nutritional evaluation. (**A**) Random forest evaluating the most important variable between well-nourished and moderate malnutrition groups. (**B**) Decision tree model for well-nourished vs. moderate malnutrition group. (**C**) Survival analysis comparing the group of well-nourished vs. moderate malnutrition group. (**D**) Random forest evaluating the most important variable between well-nourished and severe malnutrition groups. (**E**) Decision tree model for well-nourished vs. severe malnutrition groups. (**F**) Survival analysis comparing the well-nourished group with the severe malnutrition group. (**G**) Random forest evaluating the most important variable between moderate and severe malnutrition groups. (**H**) Decision tree model for moderate vs. severe malnutrition groups. (**I**). Survival analysis comparing the moderate and severe malnutrition groups.

To select the most relevant variables to predict malnutrition, we conducted a random forest analysis. We used the mean decrease in accuracy (MDA) from the random forest analysis, indicating that the gain in information or node purity is a measure of split utility. As for the well vs. moderate malnutrition group, variables such as FFMI, SMI, RF-Circ, and BCM significantly contributed to the model’s classification power (Figure 2A). This model was consistent with the decision tree model, with FFMI (cut-off less than 20) being the most crucial variable (Figure 2B), although survival analysis comparing well-nourished and moderate was not significant (*p* = 0.99) (Figure 2C). As for the well vs. severe malnutrition group, variables such as BCMI, FFMI, BCM, PA, and RF-Y-axis were the most important variables to predict severe malnutrition from the absence of malnutrition (Figure 2D). This model was confirmed by the decision tree model, with BCMI (cut-off less than 7.6 being the most important variable, Figure 2E). Moreover, the Kaplan-Meier analysis showed a significant difference in survival probability between well-nourished and severe malnutrition patients (*p* < 0.001) (Figure 2F). Finally, as for the moderate vs. severe malnutrition group, the RF-Y-axis was the most important variable to predict severe malnutrition from the moderate status (Figure 2G). This model was confirmed by the decision tree model, with the RF-Y-axis (cut-off less than 0.94) being the most important variable (Figure 2H). Moreover, the Kaplan-Meier analysis showed a significant difference in survival probability between moderate and severe malnutrition patients (*p* = 0.010) (Figure 2I).

Supplementary Figure S3 shows the confusion matrix of the decision trees. The AUC for FFMI from the well vs. moderate groups was 0.726 (cut-off of 17.7, Table 3). The AUC for BCMI from the well vs. severe groups was 0.835 (cut-off of 8.0). The AUC for the RF-Y-axis from the moderate vs. severe groups was 0.729 (cut-off of 0.94). Supplementary Tables S2 and S3 summarize the cut-off and AUC values separated by males and females.

To understand the predictive value of the variables, Supplementary Figure S4 shows the survival value for high and low values of FFMI, BCMI, and Y-axis according to their median value (medians 18.40, 9.0, and 1.05, respectively).

## 4. Discussion

In our study, we analyzed several nutritional assessment methods to define malnutrition in patients with HNC. We found that the majority of nutritional tools were well-correlated with different grades of malnutrition according to the GLIM criteria. Specifically, ultrasound methods such as the RF-Y-axis were able to discriminate between patients with moderate and severe malnutrition, which is often challenging in clinical practice. Notably, there is a significant difference in overall survival between moderately and severely malnourished patients, with the latter showing a poorer survival rate. Therefore, we highlight the usefulness of morphofunctional assessment for diagnosing malnutrition and its severity, and we propose cut-off points for different parameters to predict moderate and severe malnutrition, which is crucial in terms of survival impact.

In our study, the prevalence of malnutrition is within the range published in previous studies, ranging from 3 to 57%, depending on the combination of phenotypic and etiological criteria considered. Overall, there is a lack of unanimity in the definition of low muscle mass or the presence of inflammation [21,24,27,28,29,38]. In this study, considering the recently published Delphi consensus, we accept that all patients had inflammation to a greater or lesser degree due to being diagnosed with HNC and requiring radiotherapy (with or without chemotherapy) [30]. Therefore, to be able to compare between series, it is important to unify the definition of GLIM criteria, especially the definition used to consider a reduction in muscle mass (and its severity), as well as the application of the referred consensus to define inflammation [30].

In our results, we showed that muscle mass is a strong indicator of nutritional status and, as it has been demonstrated previously, is related to the treatment response and survival [9,10,11,12]. Therefore, it is important to detect and treat malnutrition early and to establish measures to maintain and recover muscle mass in order to optimize oncological treatment results [13,14]. To select the most relevant variables to predict malnutrition, we conducted a random forest analysis and a decision tree using variables derived from the morphofunctional assessment.

We found that for the well vs. moderate malnutrition group, FFMI was the most relevant variable, although without differences in survival between groups. For the well vs. severe malnutrition group, BCMI, and for the moderate vs. severe malnutrition group, the RF-Y-axis, were the most important variables to classify the level of malnutrition, in both cases existing a significantly different survival probability, in relation to the severe malnutrition group. The ROC curve analysis and AUC calculations for different parameters showed cut-off points to differentiate among malnutrition groups.

Nutritional ultrasound and bioelectrical impedance analysis (BIA) are simple, safe, and reproducible techniques that can be applied at the bedside and can replace reference techniques in the assessment of muscle mass. However, to date, we did not have normal ranges for specific populations such as patients with HNC. After reviewing the literature, we present the first proposal of reference values for different bioelectrical and ultrasound parameters that allow the discrimination of malnutrition patients and its severity, which, as we found, is critical as it has an impact on survival. Additionally, we provide sex-specific reference values. While these conclusions should be validated with larger studies, we highlight the relevance of these findings as they facilitate the diagnosis of malnutrition at the bedside with simple tools, and in the case of muscle mass values, they could contribute to the unification of the phenotypic criterion of muscle mass necessary to apply the GLIM criteria in this population.

There are data suggesting the prognostic relevance of muscle mass depletion in patients with HNC. Our group has recently published that RF-CSA levels measured by ultrasound lower than 2.7 cm^2^ [39] and phase angle below 5.1º and 4.8º and BCM below 28.6 kg and 17 kg in males and females, respectively, [33] are related to an increase in mortality. Sat-Muñoz D. et al. found that phase angle < 4.42º reduces survival (19.9 versus 34.4 months, *p* < 0.001) [40] and Axelsson L. et al. related phase angle < 5.95 with an increase in the five year mortality in patients with advanced HNC [41]. Lapornik et al. observed that an increase in FFMI assessed by BIA reduces the risk of mortality in their series of patients with HNC (OR: 0.88, *p* = 0.029) [42]. Although there is limited data about muscle ultrasound in HNC patients, it has been related to malnutrition in other groups of patients, such as those with post-critical SARS-CoV2 Disease, elderly home-care residents, and children with nephrotic syndrome [1,43,44,45].

Among the strengths of our study, we highlight that it is a multicenter study that includes 514 patients from 8 tertiary hospitals in the Community of Andalusia and Badajoz, so we consider it representative, and the results can be extrapolated to other centers in our area. We emphasize the relevance of morphofunctional assessment in the diagnosis of malnutrition in patients with HNC and propose reference values for different ultrasound and bioelectrical parameters useful for diagnosing malnutrition in this population. Our study has several limitations. The relatively short follow-up time does not allow for the establishment of a long-term prognosis. We must consider the intrinsic limitations of bioelectrical impedance analysis (hydration status, etc.) as well as interobserver variability in the case of ultrasound, although training was provided in the different participating centers to standardize the technique.

## 5. Conclusions

Malnutrition is highly prevalent in patients with HNC. Parameters derived from muscle mass, including bioelectrical, ultrasound, and functional measures, negatively correlate with the degree of malnutrition. Increased value of PA, BCMI, SMI, RF-CSA, RF-Y axis, and handgrip strength significantly reduce the risk of malnutrition, and we propose specific cut-off points for FFMI, SMI, BCMI, and RF-Y axis that allow diagnosing malnutrition and its severity using bedside diagnostic techniques, which is important in terms of mortality. Therefore, simple and safe techniques such as BIA and ultrasound allow for reaching an early and individualized diagnosis of malnutrition and its severity at the start of radiotherapy and should be incorporated into the daily clinical practice to identify higher-risk patients in whom to intensify nutritional treatment.

## Figures and Tables

**Table 1:** Baseline characteristics of the population of study divided by GLIM variable into three categories.

	Well-Nourished *N* = 249	Moderate *N* = 135	Severe *N* = 130	*p* Value
Demographic variables				
Age (years)	63.1 (10.8)	62.8 (11.3)	64.1 (11.5)	0.587
Sex (Male/Female)	201/42	104/29	86/38	0.165
BMI (kg/m^2^)	27.4 (4.26) ^a,b^	24.6 (4.35) ^c^	22.4 (4.84)	<0.001 ***
BIA				
PA (°)	5.47 (0.87) ^b^	5.19 (0.96) ^c^	4.72 (0.88)	<0.001 ***
SPA	−0.32 (1.10) ^a,b^	−0.54 (1.32) ^c^	−0.94 (1.34)	<0.001 ***
BCM (kg)	28.0 (5.38) ^a,b^	24.7 (5.93) ^c^	21.6 (5.59)	<0.001 ***
FM (kg)	21.6 (8.20) ^a,b^	18.8 (8.78) ^c^	15.3 (8.67)	<0.001 ***
FFMI (%)	19.6 (2.22) ^a,b^	17.9 (2.30) ^c^	16.8 (2.47)	<0.001 ***
FMI (%)	7.66 (2.88) ^a,b^	6.72 (3.18) ^c^	5.56 (3.06)	<0.001 ***
BCMI (%)	9.95 (1.65) ^a,b^	8.80 (1.78) ^c^	7.80 (1.74)	<0.001 ***
SMI (cm^2^/m^2^)	9.34 (1.45) ^a,b^	8.46 (1.52) ^c^	7.93 (1.58)	<0.001 ***
Echography exploration				
RF-CSA (cm^2^)	3.92 (1.49) ^b^	3.56 (1.23) ^c^	2.77 (1.16)	<0.001 ***
RF-CIR (cm)	8.91 (1.38) ^b^	8.62 (1.41)	8.18 (1.51)	<0.001 ***
RF-X axis (cm)	3.66 (0.65) ^b^	3.61 (0.57) ^c^	3.39 (0.72)	0.001 **
RF-Y axis (cm)	1.24 (0.47) ^a,b^	1.09 (0.32) ^c^	0.86 (0.30)	<0.001 ***
L-SAT (cm)	0.70 (0.44) ^b^	0.63 (0.37)	0.58 (0.41)	0.033
T-SAT (cm)	1.88 (4.39)	1.52 (0.64)	1.34 (0.90)	0.293
S-SAT (cm)	0.66 (0.35)	0.69 (0.34)	0.93 (4.05)	0.537
VAT (cm)	0.74 (0.47)	0.60 (0.34)	0.59 (1.25)	0.148
GAT (cm)	3.33 (4.54)	2.74 (0.99)	2.55 (1.71)	0.102
GATi (cm/m)	0.02 (0.03)	0.02 (0.01)	0.02 (0.01)	0.154
Functional measurement				
HGS max (kg)	35.5 (9.50) ^a,b^	31.9 (11.5) ^c^	28.7 (10.2)	<0.001 ***
HGS mean (kg)	33.8 (9.30) ^a,b^	30.4 (11.4) ^c^	27.2 (9.97)	<0.001 ***
TUG (s)	8.64 (4.95)	9.43 (5.48)	9.95 (4.95)	0.082
Biochemical variables				
Glucose (mg/dL)	109 (34.6)	110 (36.4)	111 (49.1)	0.920
Urea (mg/dL)	37.7 (20.3)	41.9 (32.1)	37.0 (24.0)	0.353
Creatinine (mg/dL)	0.85 (0.18) ^b^	0.81 (0.18)	0.77 (0.16)	0.002 **
Pre-albumin (mg/dL)	25.7 (6.99) ^b^	24.6 (8.39)	21.6 (7.28)	0.001 **
Total cholesterol (mg/dL)	187 (43.0)	207 (211)	179 (46.3)	0.196
Proteins (g/dL)	7.36 (4.88)	6.96 (0.75)	6.95 (0.65)	0.505
Albumin (g/dL)	4.11 (0.44) ^b^	4.00 (1.49)	3.79 (0.56)	0.010 *
hs-CRP (mg/dL)	12.3 (22.8)	22.3 (38.6)	24.4 (33.7)	0.004 **
TSH (µUI/mL)	1.57 (1.20)	2.08 (2.30)	3.61 (13.1)	0.125
HbA1c (%)	5.98 (0.75)	6.30 (1.30)	6.22 (0.98)	0.239
Clinicopathological variables				
Cancer stage				0.224
I	13 (5.58%)	12 (9.45%)	9 (7.44%)	
II	23 (9.87%)	7 (5.51%)	10 (8.26%)	
III	54 (23.2%)	25 (19.7%)	20 (16.5%)	
IVA	32 (13.7%)	14 (11.0%)	21 (17.4%)	
IVB	65 (27.9%)	50 (39.4%)	34 (28.1%)	
IVC	46 (19.7%)	19 (15.0%)	27 (22.3%)	
Complications:				<0.001 ***
No	112 (49.3%) ^a,b^	32 (27.8%)	18 (17.1%)	
Yes	115 (50.7%)	83 (72.2%)	87 (82.9%)	
Chemotherapy				0.018 *
No	128 (52.0%) ^a,b^	54 (40.0%)	50 (39.1%)	
Yes	118 (48.0%)	81 (60.0%)	78 (60.9%)	
Mortality:				<0.001 ***
No	168 (85.3%) ^b^	84 (75.7%) ^c^	58 (55.2%)	
Yes	29 (14.7%)	27 (24.3%)	47 (44.8%)	

Data are expressed as mean ± standard deviations or percentages. Groups were divided according to the GLIM criteria, being well-nourished, moderate, and severe malnutrition, the groups included in the study. Complication variables included dermatitis, dysphagia, mucositis, and asthenia. Asterisk indicates significant difference between groups, according to the ANOVA test, followed by pairwise comparisons adjusting for multiple testing (Benjamini & Hochberg method) (Chi-squared test was used for variables expressed as a percentage (*** *p* < 0.001, ** *p* < 0.01, * *p* < 0.05)). a: *p*-value, Well-nourished vs. moderate; b: *p*-value, Well-nourished vs. severe; c: *p*-value, moderate vs. severe. Abbreviations: BCM: Body cell mass; BCMI: BCM index; BMI: Body mass index; BIA: Bioelectrical Impedance Analysis; CRP: C reactive protein; FM: Fat mass; FMI: FM index; FFMI: Fat-free mass index; GAT: Global adipose tissue; GATi: GAT index; HGS: Hand grip strength; HbA1c: Glycosylated hemoglobin; PA: Phase angle; RF-CIR: circumference of quadriceps rectus femoris; RF-CSA: rectus femoris cross-sectional area; SAT: subcutaneous adipose fat of leg (L), superficial (S) and total (T) abdominal; SMI: Skeletal muscle index; SPA: Standardized phase angle; TSH: Thyroid-stimulating hormone; TUG: Timed Up & Go.

**Table 2:** Multiple logistic regression of nutritional assessment methods and the risk of malnutrition in patients with head and neck cancer.

Variables	Well-Nourished vs. Moderate			Well-Nourished vs. Severe			Moderate vs. Severe		
	OR	Lower CI 95%	Upper CI 95%	OR	Lower CI 95%	Lower CI 95%	OR	Lower CI 95%	Lower CI 95%
BIA									
PA (°)	0.77	0.58	1.01	0.48 ***	0.33	0.69	0.70 *	0.50	0.96
SPA	0.92	0.74	1.13	0.7 *	0.53	0.91	0.83	0.66	1.03
BCM (kg)	0.89 ***	0.84	0.94	0.85 ***	0.79	0.91	0.95	0.90	1.01
FM (kg)	0.98	0.95	1.01	0.98	0.94	1.01	0.99	0.95	1.03
FFMI (%)	0.70 ***	0.61	0.80	0.71 ***	0.59	0.84	0.98	0.85	1.12
FMI (%)	0.94	0.86	1.02	0.94	0.84	1.04	0.98	0.88	1.08
BCMI (%)	0.69 ***	0.58	0.81	0.6 ***	0.47	0.73	0.87	0.72	1.03
SMI (cm^2^/m^2^)	0.56 ***	0.43	0.70	0.56 ***	0.41	0.75	0.98	0.77	1.24
Echography exploration									
RF-CSA (cm^2^)	0.89	0.73	1.08	0.65 ***	0.50	0.84	0.66 ***	0.49	0.87
RF-CIR (cm)	0.93	0.77	1.77	0.81	0.65	1.01	0.94	0.75	1.16
RF-X axis (cm)	0.94	0.63	1.43	0.73	0.47	1.14	0.76	0.47	1.21
RF-Y axis (cm)	0.44 *	0.21	0.87	0.14 ***	0.05	0.36	0.16 ***	0.05	0.45
L-SAT (cm)	0.52	0.24	1.05	0.81	0.37	1.66	0.94	0.40	2.17
T-SAT (cm)	0.96	0.73	1.04	0.96	0.69	1.06	0.94	0.65	1.39
S-SAT (cm)	1.66	0.80	3.45	1.13	0.98	2.22	1.06	0.95	NA
VAT (cm)	0.6	0.31	1.11	1.03	0.69	1.42	1.14	0.82	2.05
GAT (cm)	0.89	0.70	1.03	0.97	0.79	1.06	1.05	0.84	1.33
Functional measurement									
HGS mean (kg)	0.96 ***	0.93	0.99	0.94 ***	0.9	0.97	0.99	0.95	1.02
TUG (s)	1.03	0.99	1.09	1.04	0.99	1.1	1.00	0.94	1.06

Multiple logistic regression of well-nourished vs. moderate, well-nourished vs. severe, and moderate vs. severe, and its relationship with nutritional assessment methods. All variables were adjusted for age, sex, and BMI (Categorized as BMI less than 20 for patients aged under 70 and BMI less than 22 for patients aged 70 or older) (* *p* < 0.05; *** *p* < 0.001). Abbreviations: GAT: Global adipose tissue; NA: Not applicable; RF: rectus femoris; RF-CIR: circumference of quadriceps rectus femoris; RF-CSA: rectus femoris cross-sectional area; PA: Phase Angle. PG-SGA: Patient-Generated Subjective Global Assessment; SAT: subcutaneous adipose fat of leg (L). superficial (S) and total (T) abdominal; SPA: Standardized phase angle.

**Table 3:** Predictive value of nutritional assessment method on the prediction of malnutrition in patients with HNC.

Variables	AUC	Cut-Off	Sensitivity	Specificity	*p* Value
Well-nourished vs. moderate					
FFMI	0.726	17.7	0.798	0.530	<0.001
SMI	0.724	8.6	0.769	0.541	<0.001
BCMI	0.709	8.7	0.765	0.538	<0.001
Well-nourished vs. severe					
RF-Y-axis	0.837	0.97	0.706	0.710	<0.001
FFMI	0.821	18.0	0.759	0.713	<0.001
BCMI	0.835	8.0	0.919	0.598	<0.001
BCM	0.829	22.9	0.812	0.653	<0.001
FM	0.819	16.3	0.755	0.626	<0.001
Moderate vs. severe					
RF-Y-axis	0.729	0.94	0.676	0.684	<0.001

AUC was adjusted by age, sex, and BMI. Abbreviations: AUC: Area under curve

## Data Availability

All data generated or analyzed during this study are included in this article. Further inquiries can be directed to the corresponding authors.

## Supplementary Materials

The following supporting information can be downloaded at: https://www.mdpi.com/article/10.3390/cancers16244255/s1, Figure S1: Significant correlations between body composition parameters assesed by BIVA and ultrasound nutritional evaluation, biochemical nutritional parameters, and sarcopenia in males with HNC; Figure S2: Significant correlations between body composition parameters assesed by BIVA and ultrasound nutritional evaluation, biochemical nutritional parameters, and sarcopenia in females with HNC; Figure S3: Confusion matrix of the decision trees; Figure S4: Survival value for high and low values of FFMI, BCMI, and Y-axis according to their median value.; Table S1: Baseline characteristics of the population of study under the GLIM variable; Table S2: Predictive Value of nutritional assessment method on the prediction of malnutrition in males with HNC; Table S3: Predictive Value of nutritional assessment method on the prediction of malnutrition in females with HNC.
