# Supplementary material for: Predictive Factors of the Degrees of Malnutrition According to GLIM Criteria in Head and Neck Cancer Patients: Valor Group
Source: Cancers (Basel). 2024 Dec 21;16(24):4255. doi: 10.3390/cancers16244255 (PMC11674739; doi:10.3390/cancers16244255)
Supplement: Supplementary file 1 [file cancers-16-04255-s001.zip › cancers-3356348-supplementary.pdf]

Supplementary Table S1. Baseline characteristics of the population of study under the GLIM variable.

	Well-nourished *N=249*	Moderate *N=135*	Severe *N=130*	*p* value
**Anthropometric variables**				
Weight (kg)	76.8 (13.5) [a,b]	69.0 (14.1) [c]	61.7 (15.3)	<0.001***
Weigh loss (%)	0.13 (3.70) [a,b]	6.31 (3.27) [c]	17.1 (9.90)	<0.001***
**BIA**				
Xc (Ω/m)	49.1 (9.70) [a]	52.2 (11.4)	50.1 (11.4)	0.023**
Rz (Ω/m)	515 (70.9) [a,b]	577 (93.6) [c]	610 (110)	<0.001***
**Muscle mass variables**				
MM (kg)	27.5 (5.89) [b]	25.9 (7.26) [c]	23.4 (6.70)	<0.001***
SMM (kg)	27.6 (5.92) [a,b]	25.9 (7.27) [c]	23.4 (6.70)	<0.001***
ASMM (kg)	20.9 (3.61) [a,b]	19.0 (3.91) [c]	17.1 (4.12)	<0.001***
FFM (kg)	55.2 (8.15) [a,b]	50.2 (8.61) [c]	46.5 (9.09)	<0.001***
**Water content variables**				
TBW (kg)	40.7 (6.40) [a,b]	37.0 (6.74) [c]	34.3 (7.17)	<0.001***
ECW (kg)	19.8 (3.43) [a,b]	18.5 (3.52)	18.1 (3.95)	<0.001***
ICW (kg)	28.0 (5.38) [a,b]	24.7 (5.93) [c]	21.6 (5.59)	<0.001***
NAK	1.09 (0.20) [b]	1.13 (0.28) [c]	1.26 (0.23)	<0.001***
**Metabolism and nutrition variables**				
Basal metabolism (kcal)	1560 (156) [a,b]	1420 (305)	1374 (163)	<0.001***
Hydration (%)	73.8 (2.08)	73.7 (2.59)	73.7 (2.67)	0.862
Nutrition	852 (160) [a,b]	751 (170) [c]	655 (163)	<0.001***
**Clinicopathological variables**				
Chemotherapy				0.018**
No	128 (52.0%) [a,b]	54 (40.0%)	50 (39.1%)	
Yes	118 (48.0%)	81 (60.0%)	78 (60.9%)	
Free-disease				0.085
No	135 (71.1%)	71 (70.3%)	86 (81.9%)	
Yes	55 (28.9%)	30 (29.7%)	19 (18.1%)	
Progression / Persistence				0.087
No	121 (63.7%)	61 (59.2%)	53 (50.5%)	
Yes	69 (36.3%)	42 (40.8%)	52 (49.5%)	
Palliative				<0.001***
No	160 (84.2%)	75 (72.8%)	64 (62.1%)	
Yes	30 (15.8%)	28 (27.2%)	39 (37.9%)	
Hospital admission				<0.001***
No	115 (60.5%) [a,b]	39 (37.9%)	35 (33.7%)	
Yes	75 (39.5%)	64 (62.1%)	69 (66.3%)	
ECOG:				0.001***
0	144 (64.9%) [a,b]	46 (39.0%) [c]	27 (23.3%)	
1	72 (32.4%)	63 (53.4%)	65 (56.0%)	
2	6 (2.7%)	6 (8.57%)	19 (16.4%)	
3	0 (0.00%)	1 (0.85%)	4 (4.45%)	
4	0 (0.00%)	1 (0.85%)	1 (0.86%)	

Data are expressed as mean ± standard deviations or percentage. Groups were divided by GLIM variable categorized into well-nourished, moderate and severe malnutrition. Asterisk indicates significant difference between groups, according according to the ANOVA test, followed pairwise comparisons adjusting for multiple testing (Benjamini & Hochberg method) (Chi squared test was used for variables expressed as percentage (****p*<0.001, ***p*<0.01, **p*<0.05). (****p*<0.001, ***p*<0.01, **p*<0.05). a: *p* value, Well-nourished vs moderate; b: *p* value, Well-nourished vs severe; c: *p* value, moderate vs severe.
Abbreviations: ASMM: Appendicular skeletal muscle mass; BCM: Body cell mass; BCMI: Body cell mass index; BM: Basal metabolism; ECOG: ; Eastern Cooperative Oncologic Group; ECW: Extracellular cell water; FFM: Fat free mass; FFMI: Fat free mass index; FMI: Fat mass index; MM: Mass muscle; NAK: Sodium and Potassium ratio; Rz: Resistance; SSM: Skeletal Muscle Mass; TBW: Total body water; Xc: Reactance.

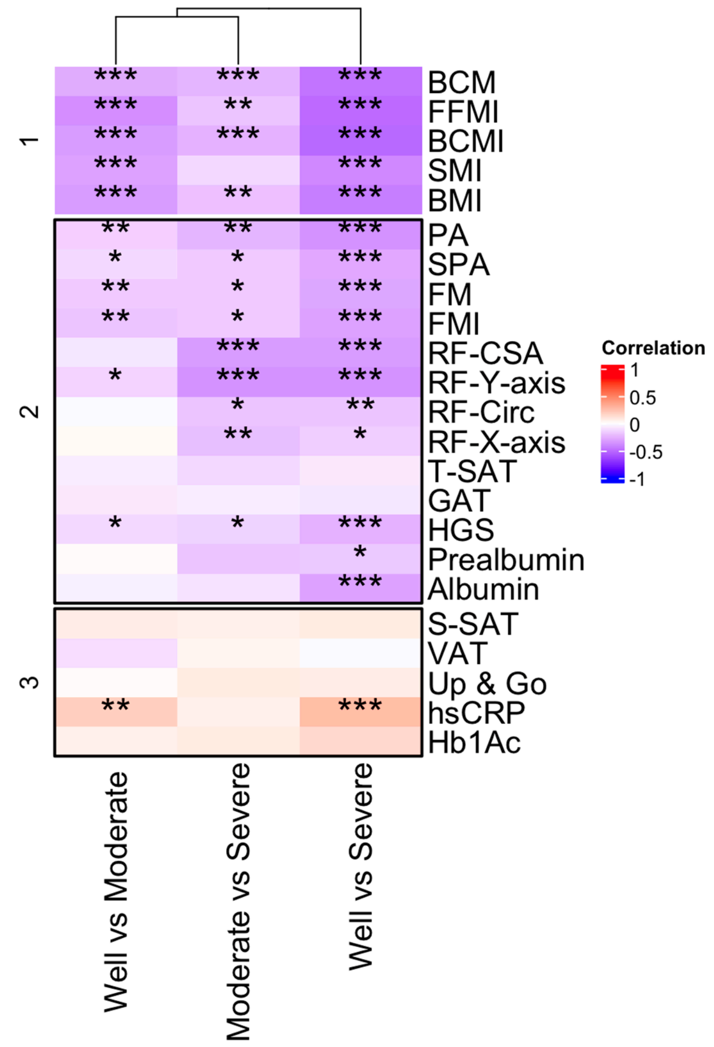

Supplementary Figure S1. Significant correlations between body composition parameters assesed by BIVA and ultrasound nutritional evaluation, biochemical nutritional parameters, and sarcopenia in males with HNC.

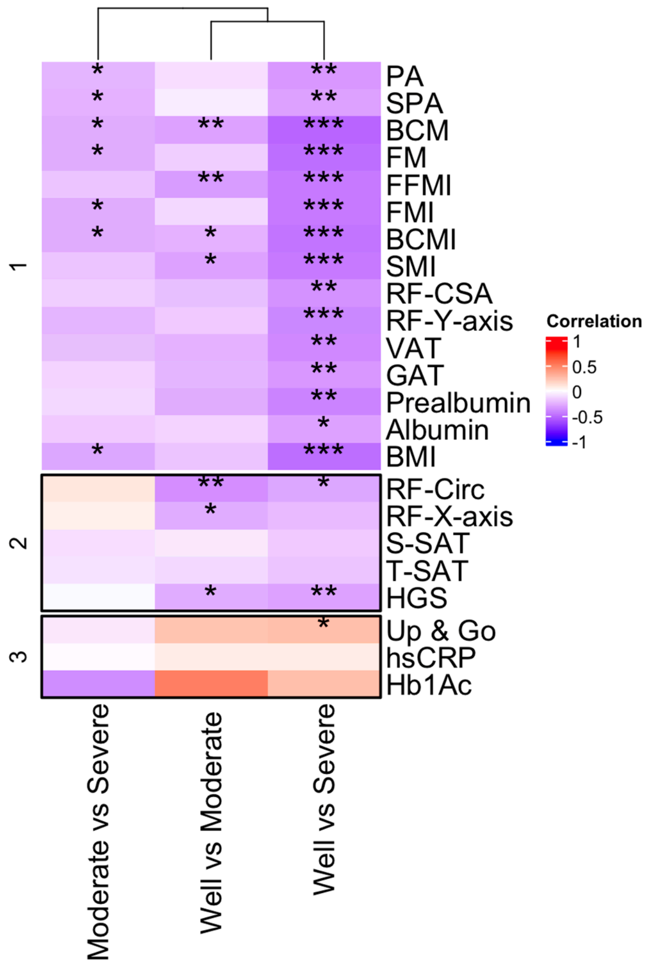

Supplementary Figure S2. Significant correlations between body composition parameters assesed by BIVA and ultrasound nutritional evaluation, biochemical nutritional parameters, and sarcopenia in females with HNC.

		Predicted	
		Well-nourished	Moderate
Observed	Well-nourished	**61**	10
	Moderate	33	**145**

Accuracy CI 95%: 0.8327 (0.774 – 0.872)
Kappa: 0.614
P<0.001
AUC: 0.610

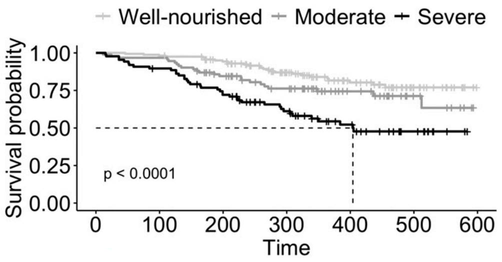

		Predicted	
		Well-nourished	Severe
Observed	Well-nourished	**65**	14
	Severe	23	**141**

Accuracy CI 95%: 0.848 (0.796 – 0.890)
Kappa: 0. 663
P<0.001
AUC: 0.802

		Predicted	
		Moderate	Severe
Observed	Moderate	**76**	15
	Severe	18	**73**

Accuracy CI 95%: 0.819 (0.755 – 0.972)
Kappa: 0. 516
P<0.001
AUC: 0.282

Supplementary Figure S3. Confusion matrix of the decision trees.

Supplementary Table S2. Predictive Value of nutritional assessment method on the prediction of malnutrition in males with HNC

Variables	AUC	Cut-off	Sensitivity	Specificity	*p* value
Well-nourished vs moderate					
FFMI	0.734	18.0	0.856	0.490	<0.001
SMI	0.738	8.6	0.896	0.432	<0.001
BCMI	0.714	8.5	0.871	0.441	<0.001
Well-nourished vs severe					
RF-Y-axis	0.827	1.07	0.672	0.726	0<.001
FFMI	0.823	18.1	0.835	0.667	<0.001
BCMI	0.831	8.7	0.840	0.667	<0.001
BCM	0.816	24.3	0.838	0.593	<0.001
FM	0.823	16.4	0.729	0.635	<0.001
Moderate vs severe					
RF-Y-axis	0.723	0.94	0.779	0.575	<0.001

AUC was adjusted for age and BMI. **Abbreviations**: AUC: Area under curve; RF-CIR: circumference of quadriceps rectus femoris; RF-CSA: rectus femoris cross-sectional area.

Supplementary Table S3. Predictive Value of nutritional assessment method on the prediction of malnutrition in females with HNC

Variables	AUC	Cut-off	Sensitivity	Specificity	*p* value
Well-nourished vs moderate					
FFMI	0.741	16.4	0.775	0.571	0.006
SMI	0.737	7.1	0.625	0.724	0.012
BCMI	0.701	8.0	0.732	0.607	0.028
Well-nourished vs severe					
RF-Y-axis	0.836	0.86	0.676	0.750	<0.001
FFMI	0.797	16.2	0.825	0.710	<0.001
BCMI	0.809	8.0	0.732	0.789	<0.001
BCM	0.836	19.8	0.731	0.763	<0.001
FM	0.823	21.0	0.634	0.921	<0.001
Moderate vs severe					
RF-Y-axis	0.694	0.72	0.840	0.417	0.120

AUC is adjusted for age and BMI **Abbreviations**: AUC: Area under curve; RF-CIR: circumference of quadriceps rectus femoris; RF-CSA: rectus femoris cross-sectional area.

		Predicted	
		Well-nourished	Moderate
Observed	Well-nourished	**61**	10
	Moderate	33	**145**

Accuracy CI 95%: 0.8327 (0.774 – 0.872)
Kappa: 0.614
P<0.001
AUC: 0.610

		Predicted	
		Well-nourished	Severe
Observed	Well-nourished	**65**	14
	Severe	23	**141**

Accuracy CI 95%: 0.848 (0.796 – 0.890)
Kappa: 0. 663
P<0.001
AUC: 0.802

		Predicted	
		Moderate	Severe
Observed	Moderate	**76**	15
	Severe	18	**73**

Accuracy CI 95%: 0.819 (0.755 – 0.972)
Kappa: 0. 516
P<0.001
AUC: 0.282

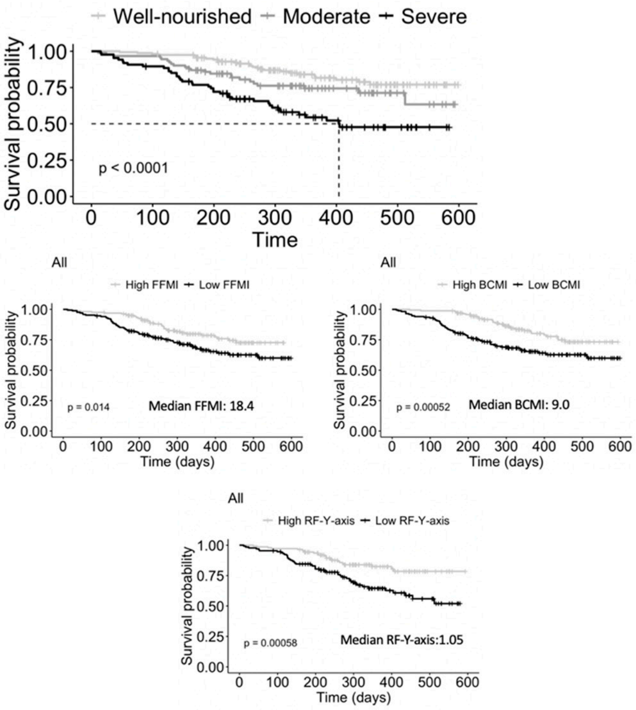

Supplementary Figure S4. Survival value for high and low values of FFMI, BCMI, and Y-axis according to their median value.
